# Have we bothered to ask? Exploration of the attitude of teachers toward participating in inclusive education research

**DOI:** 10.1186/s13104-024-06755-2

**Published:** 2024-03-28

**Authors:** Maxwell Peprah Opoku, William Nketsia, Mprah Kwadwo Wisdom, Michael Amponteng

**Affiliations:** 1 Department of Special and Gifted Education, United Arab Emirates University, Al Ain, United Arab Emirates; 2 School of Education, Western Sydney University, Sydney, Australia; 3 Centre for Disability and Rehabilitation Studies, Department of Health Promotion and Disability Studies, Kwame Nkrumah University of Science and Technology, Kumasi, Ghana

**Keywords:** Inclusive education, Inclusive education research, Teachers, Refusal, Policy, Practices, Ghana

## Abstract

**Objective:**

The importance of research cannot be overemphasized. Research findings serve as a guide for the enactment of development policies and legislation. However, not all members of the target population willingly participate in a study. The current study explored the reasons why some individuals refused to partake in inclusive education research in a developing country, Ghana. The journaling helped to capture the voices of 87 participants who refused to take part in a larger inclusive education survey study.

**Results:**

The study found that the participants did not take part in the research because of reasons such as lack of financial gain, bad experience with previous research, lack of direct benefit, and lack of time. The findings of the study and its implication for policymaking in Ghana and research studies in sub-Saharan Africa are discussed.

## Introduction

There has been discussion on the need for a close link between theory and practice in the implementation of policies such as inclusive education [1]. This means that decisions on reforms or steps to improve practices will be based on information made available through research. Indeed, research has been described as an important bedrock of development as it provides direction to improve lives and practices. Research has the potential to enable the gathering of information, development of new insights, understand challenges affecting practices, and develop policy guidelines [2]. Consequently, universities have been at the forefront of research and they make their findings known to industry players at workshops, seminars and advocacy to inform practices [2–4]. This has seen governments, industries and individuals channeling resources into researches to get new insights and making recommendation to advance lives of individuals [2] such as those living with disabilities in societies.

In this study, inclusive education is defined narrowly as creating opportunities for students with mild disabilities, such as physical, visual, hearing, and behavioral disabilities, to participate in regular schools in their communities [5]. The publication of the Salamanca conference report was hailed as the first attempt toward advocacy for the implementation of inclusive education [6]. Further to this, was the promulgation of the Convention on the Rights of Persons with Disabilities [7] which has been widely accepted as a key milestone towards achieving the goal of educating all students in one classroom. Research continue to be encouraged, in almost all countries, to help bridge the gap between policy intentions and actual practices in classroom.

Although gains have been made in areas of policy reformation and acceptance of the policy in schools [5], there is still a significant gap between policy and practices [8–10]. The worse affected areas are developing contexts in sub-Saharan Africa where significant barriers affect the successful implementation of inclusive education in schools [11–15]. However, the quality of information from stakeholders such as teachers is important to ensure that the right policy decision is being made by policymakers. To our best of knowledge, only one study has documented the factors explaining one’s decision for participating or refusal to take part in a study. A rare example is a health research conducted in Malawi by Mfutso-Bengo et al. [16]. The study used focus group discussion to document the experiences of participants who had refused to take part in an empirical research. It emerged that researchers did not follow traditional customs, participants were afraid of strangers; superstition and negative experience in previous research, improper informed consent process, poor timing and inability of the researchers to communicate the direct benefit of the research to the participants. However, such study is unavailable in the field of inclusive education. The purpose of this study was to document the perception of teachers towards participating in research on inclusive education for children with disabilities.

## Methods

The researchers conveniently recruited participants from five districts in a region in Ghana between January 2018 to September 2018, to participate in a larger study on intention towards implementation of inclusive education [12–15]. Out of 897 teachers contacted to participate in the large study related to inclusive education, 496 agreed and took part in the study. However, 401 did not take part in the study. As this study aimed at exploring the main reasons for not participating in the large study, the first inclusion criterion was consideration of teachers who refused to participate in the larger study and were willing to explain their non-response or non-participation. One hundred and fifty-seven did not give a reason for their exclusion from the large study and thus were excluded from this study. In addition, the researcher could not get the chance to interact with 128 participants and could only engage 116 participants. The second inclusion criterion was to include participants who authenticated their conversations with the first author. Out of 116 participants, 87 participants approved the content with their reasons for not participating in the large study while 29 participants were unavailable to comment despite being given prior notice by the first author.

After obtaining the necessary permission from institutional review board for the larger study (University of Tasmania [H0016994] and Special Education Division of Ghana Education Service [se.183/104]), the researcher approached the schools to seek permission to conduct this study. When permission was granted by head-teachers of the schools, they asked one of the teachers to assist the researcher with the data collection. At the staff common rooms of the schools, the researcher spoke to all the teachers while the teacher supporting the data collection went around to give the questionnaire to their colleagues interested in completing the survey.

The researcher observed the process and approached the teachers who refused to take part in this larger study. The researcher engaged them in a friendly conversation and asked them if they would be interested to take part in a conversation. The observational checklist was shown to the participants and informed consent was obtained from the participants before they were included in the study. The researcher ticked the reason given by the participants. The objectives of the study were discussed with the participants. They were also informed about data safety, privacy, and use without harming personal identity. The questions that guided the discussion were: Why would not you participate in the large study? If responses were unclear, the researcher followed up with additional questions for clarification.

The data was analysed by performing the content analysis and follow step outlined by Burns [17]. The narratives were reconstructed based on these themes to portray their views, opinions, and reasons for not being interested to participate in the large study. Consequently, systematic conclusions were drawn and developed into this final report.

## Findings

### Immaterial to the needs of participants

One of the themes that emerged was that the study and its focus was immaterial to the needs of prospective participants. Most were of the view that the study being conducted does not address their concerns and not a matter of priority. They think the researcher was conducting a study that is out of touch with realities in Ghanaian societies. Some alleged that the study and its focus suits a western society where there are abundant financial and material resources to cater for the needs of all students in schools. It would, therefore, not benefit the society since the country is not in a position to open regular schools to every student. The researcher informed the prospective participants that the study intended to assess only intentions and their preparedness to teach students with disabilities even if they were not teaching students who were focus of the study. Most of them directed the researcher to go to special schools and administer the questionnaire to teachers teaching students with disabilities who were the focus of the study.

*The research you are doing, won’t solve our problems. We have pressing needs and what you doing is not intended solve our issues;*

*The best people to respond to your questionnaires are in special schools. There is one at the outskirt of town; I think it’s best to collect data there.*

### Lack of time and disruptions to activities

Another theme that emerged was lack of time and others see the research as disruption to academic activities. Some head teachers, especially, those from private schools informed the researcher that their teachers would not have time to participate in this study. Some said their teachers are supposed to supervise students all day and thus, will not make time to respond to the survey questionnaire. Others also said that their teachers have so many things doing at that time and cannot complete the survey questionnaire:

*The teachers are busy and I’m afraid, they can’t participate in this study. One of the duties of teachers is to keep an eye on students all day. We close late and they are exhausted by the time they get home.*

At the individual level, some teachers mentioned the fact that they would not have time to take part in the study. They said they have huge teaching loads which give them little time to partake in our research activities. Others said that they teach in different schools which would make it difficult for them to make time for the study.

### Demand for money

The most common theme was demand for money by most of the none-response participants. Most teachers requested that they are paid before they could participate in the study. Most were of the view that the researcher was studying outside Ghana and as such, has been given money to undertake the study. Some also said that what they were doing would bring them money and if the researcher wanted them to participate in the study, they have to be paid for the lost time. When alerted that the study is a requirement for a degree and no money has been given to the researcher to give to all participants, they declined to take part in the study:

*I can’t leave what I’m doing and answer all these questions for you. You won’t come here to collect this data if you have not been given to this. Pay me then would stop the marking and also help you. If not, I can’t take part in your research.*

### Lack of implementation of previous research

Another reason given by most prospective participants for their non-participation in this study was the lack of implementation of previous research. Most were of the view that there were numerous researches in the shelves of universities and colleges of education which has gone unimplemented. Because of this, they thought it would be unnecessary for them to participate in new research. To them, it makes no sense to participate in research whose findings would not be implemented:

*I have made my mind not to take part in any research. It’s a complete waste of time because they add nothing to our development. Go to the universities, we have been doing research since independence and nothing has been achieved in this country.*

## Discussion

Once research is placed at the forefront of our development, individuals would participate in inclusive education research without thinking about directs benefit but would see it a venture that would benefit the entire society. The findings suggest that there are some key stakeholders in education who do not see the benefit of inclusive education research. The study finding is inconsistent with a previous study in Malawi which found cultural barriers as reasons for refusal of participants to contribute to medical research [16].

The findings of the study have implication for policymaking in Ghana and research studies in sub-Saharan Africa. First, universities and tertiary institutions could consider encouraging students to share their research findings in inclusive education with the society. This would enable society and even policymakers come to terms with problems derailing the implementation of inclusive education. Second, universities and academics could take the lead with respect to the sensitization of society about importance of inclusive education research and the need for participation of all teachers. They could conscientise individuals to understand that research is not a ‘money making’ venture but a project to find solution to common problems in the society. Therefore, participation of individuals is key to enable holistic understanding of systemic problems. Third, policymakers could consider working hand-in-hand with academics and consult them on inclusive education research findings during decision-making.

### Study limitations

The findings of this study have to be interpreted cautiously because of the following limitations. Firstly, the study was an observation and field reflection. Thus, comments may have been lost since the notes were made after the encounter with prospective participants. Additionally, the researchers randomly selected schools in five districts in one region. It is possible that other participants may have different perspective or would have participated in the study. It is recommended that future studies are conducted in other regions (or similar con-texts) and gather in-depth understanding of other professionals such as, those in the health sector, judiciary or civil servants.

## Data Availability

The datasets used and/or analysed during the current study are available from the corresponding author on reasonable request.
